# Maternal *schistosomiasis* impairs offspring Interleukin-4 production and B cell expansion

**DOI:** 10.1371/journal.ppat.1009260

**Published:** 2021-02-01

**Authors:** Diana Cortés-Selva, Lisa Gibbs, Andrew Ready, H. Atakan Ekiz, Ryan O’Connell, Bartek Rajwa, Keke C. Fairfax

**Affiliations:** 1 Department of Pathology, Division of Microbiology and Immunology, University of Utah, Salt Lake City Utah, United States of America; 2 Department of Comparative Pathobiology, College of Veterinary Medicine, Purdue University, West Lafayette Indiana, United States of America; 3 Bindley Bioscience Center, Purdue University, West Lafayette Indiana, United States of America; University of Pennsylvania School of Veterinary Medicine, UNITED STATES

## Abstract

Epidemiological studies have identified a correlation between maternal helminth infections and reduced immunity to some early childhood vaccinations, but the cellular basis for this is poorly understood. Here, we investigated the effects of maternal *Schistosoma mansoni* infection on steady-state offspring immunity, as well as immunity induced by a commercial tetanus/diphtheria vaccine using a dual IL-4 reporter mouse model of maternal schistosomiasis. We demonstrate that offspring born to *S*. *mansoni* infected mothers have reduced circulating plasma cells and peripheral lymph node follicular dendritic cells at steady state. These reductions correlate with reduced production of IL-4 by iNKT cells, the cellular source of IL-4 in the peripheral lymph node during early life. These defects in follicular dendritic cells and IL-4 production were maintained long-term with reduced secretion of IL-4 in the germinal center and reduced generation of TFH, memory B, and memory T cells in response to immunization with tetanus/diphtheria. Using single-cell RNASeq following tetanus/diphtheria immunization of offspring, we identified a defect in cell-cycle and cell-proliferation pathways in addition to a reduction in Ebf-1, a key B-cell transcription factor, in the majority of follicular B cells. These reductions are dependent on the presence of egg antigens in the mother, as offspring born to single-sex infected mothers do not have these transcriptional defects. These data indicate that maternal schistosomiasis leads to long-term defects in antigen-induced cellular immunity, and for the first time provide key mechanistic insight into the factors regulating reduced immunity in offspring born to *S*. *mansoni* infected mothers.

## Author summary

Maternal helminth infections are a global public health concern and correlate with altered infant immune responses to some childhood immunizations, but a mechanistic understanding of how maternal helminth infection alters the cellular immune responses of offspring is lacking. Here we establish a model of maternal *Schistosoma mansoni* infection in dual IL-4 reporter mice. We find that offspring born to mothers infected with *S*. *mansoni* have impaired production of IL-4 during homeostasis, and following immunization with a Tetanus-Diphtheria vaccine. We identified that iNKT cells are the dominant source of IL-4 during early life homeostasis, and that diminished IL-4 production was associated with both reduced B cell and follicular dendritic cell responses. These defects were maintained long-term, affecting memory B and T cell responses. Single-cell RNASeq analysis of immunized offspring identified egg antigen-dependent reductions in B-cell cell cycle and proliferation-related genes. These data reveal that maternal infection leads to long-lasting defects in the cellular responses to heterologous antigens and provide vital insight into the influence of maternal infection on offspring immunity.

## Introduction

Schistosomiasis is an infectious disease caused by trematode parasites of the genus Schistosoma, with *S*. *mansoni*, *S*. *haematobium*, and *S*. *japonicum* causing the most human morbidity [1]. This disease affects an estimated 779 million people who are at risk, and 207 million people infected annually [2], with the highest prevalence in adolescents and young adults (10 to 24 years of age) [3]. This situation leads to a unique impact on women of reproductive age, with 40 million women infected annually. Human studies on schistosomiasis and pregnancy have previously established a link between maternal infection and low birth weight, as well as premature birth and intrauterine growth restriction [4–6]. Moreover, the examination of the effects of maternal schistosomiasis on the response of offspring to heterologous antigens has demonstrated impaired responses in childhood-vaccine induced immunity, raising the concern that a mother’s parasitic status during pregnancy might render early childhood immunization ineffective for years and even decades post-immunization [7]. Decreased responses to bacillus Calmette-Guérin (BCG) have been shown in children sensitized *in utero* to *Schistosoma haematobium* [8]. Furthermore, children born to mothers infected with *S*. *mansoni* presented lower anti-measles antibody levels [7] at 2 years of age. Altogether, these data suggest that populations where schistosomiasis is endemic, are vulnerable to vaccine failure, and consequently, susceptible to deaths due to preventable infectious diseases. Endemic regions may require an adjusted immunization regimen in order to ensure optimal protection against diseases, but mechanistic studies are needed to examine this.

Similar to the human data, mouse studies have shown reduced protection from BCG vaccine against *Mycobacterium tuberculosis* challenge in mice infected with *S*. *mansoni* [9]. Further, infection during gestation has shown increased susceptibility of offspring to subsequent *S*. *mansoni* infection [10]. Many studies have described a state of hyporesponsiveness to homologous antigens that has been attributed, in part, to immune factors acquired from the mother such as IL-10, Igκ and/or previous antigen contact [11,12]. Previous sensitization of murine offspring to antigens from mothers infected with other parasitic infections such as *Wuchereria bancrofti* and/or *Plasmodium falciparum* have shown a Th-2 biased response (higher production of *il-4* mRNA and IgE antibody) to diverse antigens when compared to mice from uninfected mothers [13]. However, the effects of schistosomiasis during pregnancy on the cellular immune response in offspring during homeostasis, and when challenged with heterologous antigens remain poorly defined [14]. *S*. *mansoni* infection elicits host responses that are similar in humans and mice, so the murine model of schistosomiasis is useful to determine the mechanisms by which prenatal infections induce diminished postnatal immune responses.

In this study, we aimed to define the effects of perinatal *S*. *mansoni* infection on offspring homeostatic immunity during early life and determine whether maternal infection causes a deleterious or beneficial effect in response to immunization with heterologous antigens. We have established a novel experimental murine model of maternal schistosomiasis in IL-4 dual reporter mice. Our data from this model demonstrate that maternal schistosomiasis reduces steady-state iNKT and CD4-T cell production of IL-4. This reduction correlates with reduced follicular dendritic cells (FDCs) and circulating plasma and memory B cells at steady state. Additionally, the cellular responses to immunization with commercially licensed tetanus/diphtheria vaccine are diminished following primary immunization, leading to a defect in memory T follicular helper (TFH) precursors. Single cell RNAseq analysis revealed reductions in multiple cell cycle/proliferation genes, as well as the critical B cell transcription factor Ebf-1. Thus, *in utero* exposure to *S*. *mansoni* antigens induces long-lived modulation of the development of humoral and cellular responses via suppression of IL-4 production, and the B cell-stromal cell axis.

## Results

### Maternal *Schistosoma mansoni* infection results in *in utero* egg antigen sensitization and a reduction in the B cell-stromal cell axis

**Fig 1:**
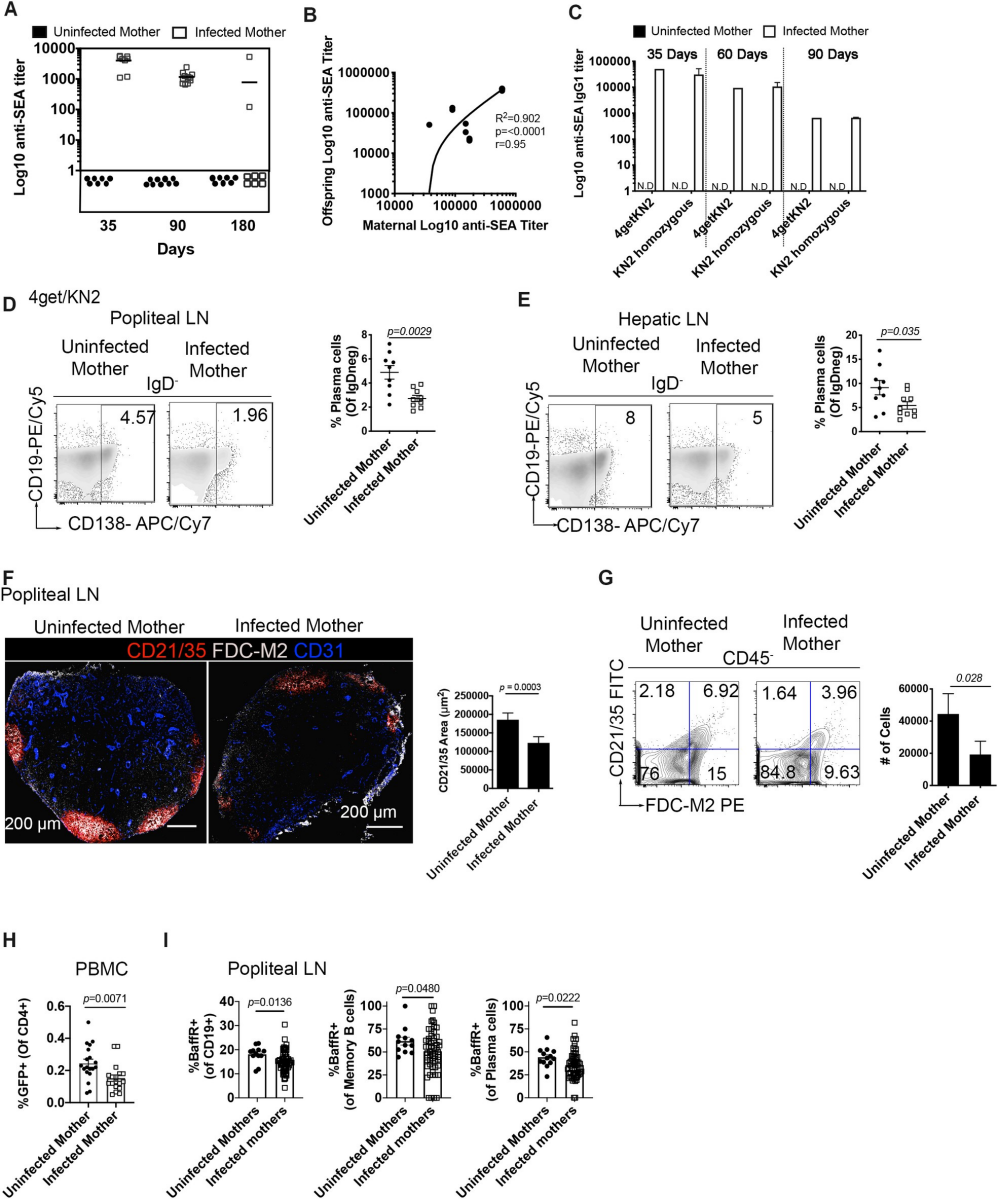
Maternal *S*. *mansoni* infection leads to a humoral anti-SEA response in offspring and a reduction in follicular dendritic cells. (A) anti- *Schistosoma mansoni* egg antigen (SEA) specific IgG1 antibody titers in serum of naïve 4get/KN2 mice born to infected or uninfected mothers. Each point represents an individual mouse. (B) Analysis of the correlation between maternal and offspring anti-SEA specific IgG1. (C) Anti-SEA IgG1 titers in naïve KN2 homozygous and 4get/KN2 mice born to infected and uninfected mothers at 35, 60, and 90 days of age. (D-E) Flow cytometry plots of plasma cells (IgD^-^CD19^+/-^CD138^+^) in popliteal and hepatic lymph nodes from naïve 4get/KN2 pups born to infected and uninfected mothers. (F) Tile confocal imaging of naïve popliteal lymph nodes of 4get/KN2 mice at 28–35 days of age, scale bar: 200 μm. Sections were stained for CD21/35 (red) FDC-M2 (gray) and CD31 (blue) with the respective CD21/35 area quantification. (G) Flow cytometry analysis of FDCs (CD21/35^+^FDC-M2^+^) gated from CD45^-^ with the total number of FDC in popliteal lymph node from pups 28–35 days of age. (H) Frequency of CD4^+^ GFP^+^ in the blood of naïve 4get/KN2 mice. (I) Flow cytometry analysis of the expression of BaffR in CD19^+^ B cells, memory B cells and plasma cells. Flow plots were concatenated samples, with 3–6 mice per group and representative of six independent experiments. Confocal microscopy data is representative of n >3 mice per group from two biologically independent experiments. Statistical significance was calculated by unpaired Student's t-test. Error bar denotes mean ± SEM. Correlation analysis was calculated by Pearson correlation coefficient.

We infected 4get homozygous (IL-4 reporter mice on a BALB/c background [15]) female mice with a low dose of *S*. *mansoni* (35 cercariae) as described in Materials and Methods. At five to six-weeks post infection (the timepoint where egg production begins), they were paired with a naïve KN2 homozygous (IL-4 deficient [16]) male. At 6 weeks post infection, females were tested for antibodies specific to Schistosoma egg antigen (SEA) to confirm patent infection. Age-matched mixed-gender pups from infected and control uninfected mothers were weaned at 28 days to maximize survival and lactation period. At 28–35 days old, pups were sacrificed for steady-state studies (S1 Fig). Data presented throughout are representative of offspring born to mothers across the spectrum of infection (acute-chronic). SEA specific antibodies in pups from infected and uninfected mothers were tested at 35, 90, and 180 days of age (Fig 1A). As expected, pups from mock-infected females (controls) showed no antigen-specific response to SEA at any of the timepoints. In contrast, pups from infected mothers exhibited an anti- SEA IgG1 antibody response at 35, 90, and 180 days of age. In some cases, at 180 days of age, some of the pups exhibited no detectable anti-SEA IgG1 (Fig 1A), indicating heterogeneity in the humoral response to egg antigens. As expected, we found that at 28–35 days of age, offspring anti-SEA IgG1 titers correlate with maternal titers (Fig 1B, p<0.0001 R^2^ = 0.902). Next, we wondered if the antigen-specific antibody was maternally derived or self-derived. For this, we mated 4get/KN2 (infected or uninfected) females and KN2 homozygous males to obtain KN2 homozygous or 4get/KN2 littermate offspring. Since KN2 homozygous mice are IL-4 deficient, they should have a diminished ability to switch to IgG1 in response to SEA [17]. We observed that at 35, 60, and 90 days of age, KN2 homozygous and 4get/KN2 offspring had detectable anti-SEA IgG1 antibodies and that those titers were similar between KN2 and 4get/KN2 animals (Fig 1C).

Exposure *in utero* to *S*. *mansoni* egg antigens is correlated to a significantly diminished bulk plasma cell population at steady-state in both popliteal (Fig 1D) and hepatic lymph nodes (Fig 1E), with a 42% reduction of plasma cells in popliteal lymph nodes and a 60% reduction of total plasma cells in hepatic lymph nodes. Follicular dendritic cells (FDC) trap immunocomplexes via Fc and Complement receptors [18]. Previous studies have established that FDCs serve as sites of B cell antigen capture [19], and that persisting antigens trapped by FDC induce somatic hypermutation [20]. Since we found that pups born to infected mothers have reduced plasma cells in lymph nodes, we sought to determine whether this is accompanied by modifications in the stromal cell population of FDCs (defined as CD21/35, complement receptor 2 and 1, respectively and FDC-M2 positive) via tile confocal microscopy. Indeed, in pups from infected mothers, a marked decrease of intensity in the markers for FDCs is observed (Fig 1F). In addition to reduced intensity of CD21/35 and FDC-M2 in naïve peripheral lymph nodes, we also observed reduced area of CD21/35 FDCM2 double-positive cells. To confirm the confocal tile scan results, we analyzed lymph nodes by flow cytometry and confirmed that offspring born to *S*. *mansoni* infected mothers have reduced frequency and total cell number of FDC in peripheral lymph nodes (Fig 1G). We then analyzed the lymphocyte compartment to determine if B or CD4^+^ T cell development is altered by maternal *S*. *mansoni* infection. We found no difference in the absolute numbers of CD19^+^ cells in peripheral lymph nodes. Our recent work demonstrated a role for IL-4 in the homeostatic maintenance of FDCs [21], so we analyzed IL-4 competent Th2 CD4^+^ T cells (GFP^+^) and found a reduction in Th2 cells in the blood (PBMCs Fig 1H). The Baff-BaffR signaling pathway is known to be critical for long-term B cell survival, so we measured BaffR expression via FACS and found reduced BaffR in bulk CD19^+^, plasma, and memory B cells (Fig 1I). These data suggest that egg antigen sensitization alters two critical pathways that provide survival signals to B cells, BaffR, and immune complex presentation.

### Invariant NK T cells are the source of IL-4 in the peripheral lymph node at steady state

**Fig 2:**
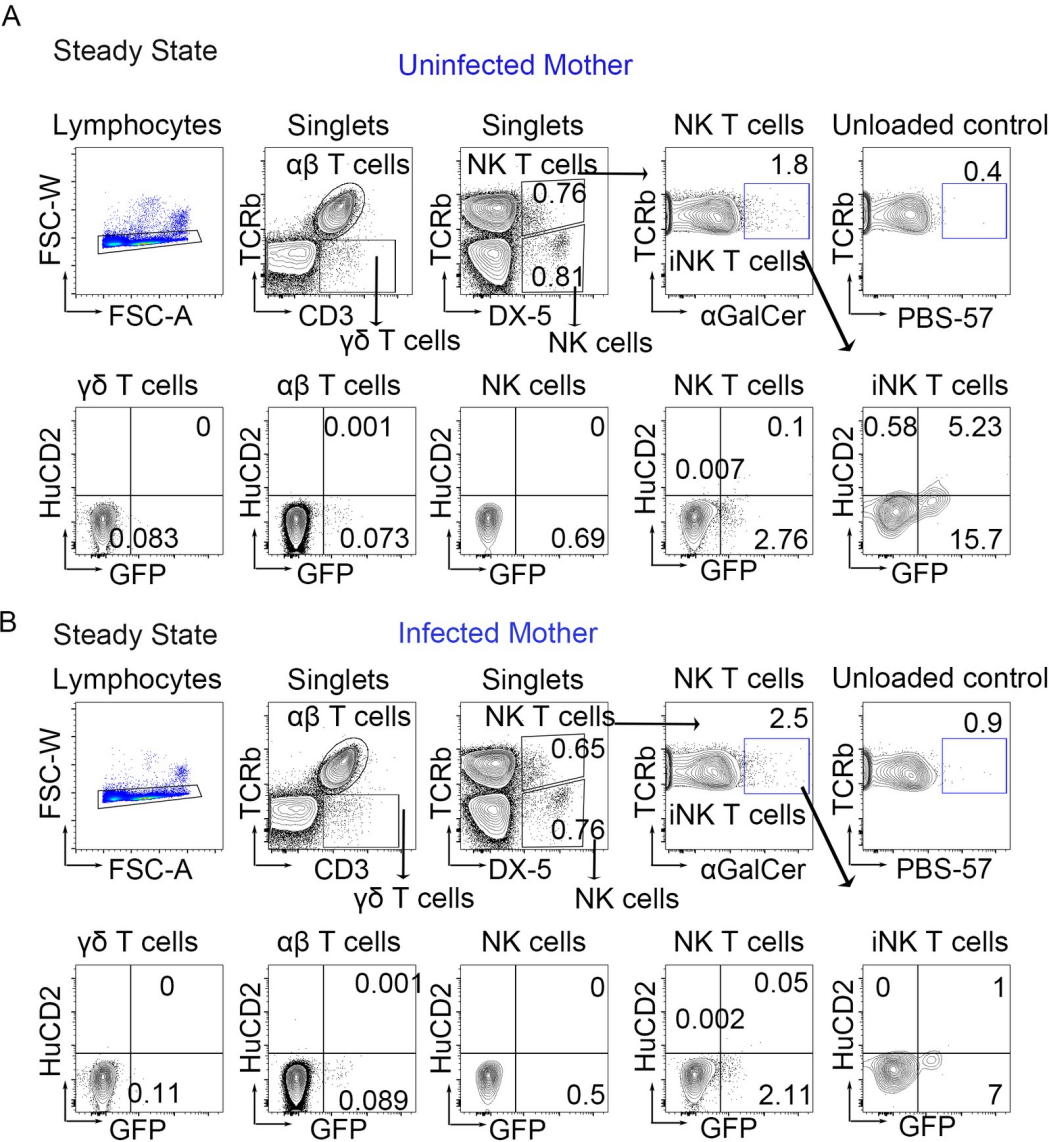
iNKT cells are the main cellular source of IL-4 in peripheral lymph nodes at steady state, and maternal *S*. *mansoni* infection reduces their secretion of IL-4. Gating strategy in popliteal lymph nodes for invariant NKT cell (αGalCer^+^TCRαβ^+^ DX-5^+^), αβ T cells (TCRβ^+^CD3^+^), γδT cells (TCRβ^-^CD3^+^), NK T cells (TCRβ^+^DX-5^+^), NK cells (TCRβ^-^DX-5^+^) with respective staining control (unloaded tetramer) analyzed by flow cytometry in (A) pups from uninfected mother and (B) pups from infected mother at 28–35 days of age. Flow plots are concatenated from >4 mice per group with two biologically independent experiments.

Since we observed reduced Th2 (GFP^+^) CD4 T-cells at steady-state in the blood of pups from infected mothers in comparison to control pups, we analyzed a diverse spectrum of cells, including γδ T cells, αβ T cells, NK cells, NKT cells and iNKT cells to look for the steady-state secretion of IL-4 (marked by HuCD2). We observed very little secretion of IL-4 in any cell populations except for invariant NKT-cells (Fig 2A). Moreover, there was a marked decrease in both transcription and secretion of IL-4 in the lymph nodes of offspring from infected mothers, with a 5-fold reduction in IL-4 secreting iNKT cells from pups that were antenatally exposed to SEA antigens (Fig 2B). These data suggest that steady-state IL-4 production is attenuated by antenatal *S*. *mansoni* antigen exposure and establishes that iNKT cells are the homeostatic source of lymph node IL-4 during early life.

### Offspring born to infected mothers have reduced Th2 development following primary immunization with tetanus/diphtheria

**Fig 3:**
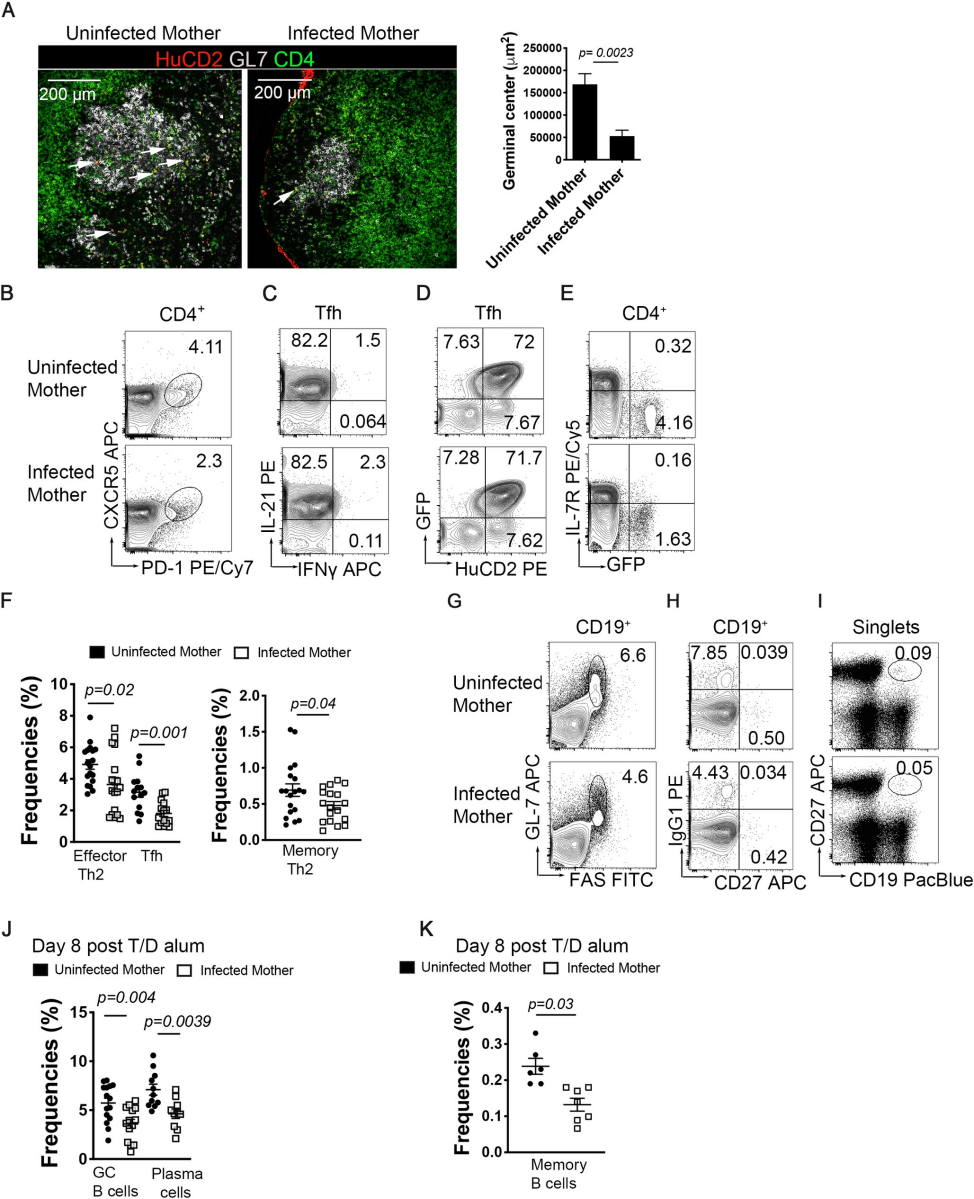
Maternal schistosomiasis leads to reduced TFH cells and IL-4 secretion in response to Tetanus/Diphtheria immunization. 4get/KN2 offspring born to *S*. *mansoni* infected mothers or uninfected control mothers were immunized in the rear footpad with 1/10^th^ of the human dose of an aluminum adjuvanted Tetanus/Diphtheria vaccine. (A) Tile confocal imaging of 10-micron cryo-sections of popliteal lymph nodes at day-8 post-immunization, stained for HuCD2 (red), GL-7 (gray), and CD4 (green). Scale bar: 200 μm and respective quantitation of germinal center area. Flow cytometry analysis from popliteal lymph nodes of (B) T follicular helper cells (PD-1^+^CXCR5^+^, gated from CD4^+^), (C) intracellular IL-21 and INF-gamma co-production by TFH (D) IL-4 production and secretion by TFH (E) Th2 effector (GFP^+^IL-7R^-^) and Th2 memory (GFP^+^IL-7R^+^). (F) Frequencies of effector Th2, memory Th2 and TFH cells at 8 days post immunization. (G) Flow cytometry of germinal center B cells (CD19^+^GL-7^+^FAS^+^) (H) Memory IgG1^+^CD27^+^ B cells (I) Bulk memory B cells (CD27^+^CD19^+^) (J) Cell frequencies of GC B cells and plasma cells (K) Frequency of bulk memory B cell in popliteal lymph node. Confocal microscopy images are representative of four independent experiments. Flow plots are concatenated from n >3 mice from at least two biologically independent experiments. Statistical significance was calculated using Student’s t-test.

Having confirmed that maternal infection induces changes in the immune response in offspring at steady-state, we wondered whether the immune response to heterologous antigens could be affected by *S*. *mansoni* maternal infection. We immunized 28-35-day-old 4get/KN2 pups subcutaneously (rear footpad injection) from infected and uninfected mothers with ~1/10^th^ of the human dose of adjuvanted Tetanus/Diphtheria vaccine [21]. The goal of these studies is to understand early life immune responses to immunization with clinically relevant foreign antigens. We have timed immunization as closely to the time of weaning as possible to mimic the timing of early childhood immunization. The timing of immunization was further influenced by published observations that concentration of maternal-derived Immunoglobulin start to wane at 4 weeks of age until gradually decreasing to adult levels at 6 weeks of age [12]. At eight days post-immunization, we observed that pups from infected mothers exhibited reduced germinal centers in popliteal lymph nodes as well as reduced IL-4 production (marked by HuCD2) in the draining lymph node (Fig 3A). Moreover, offspring born to infected mothers presented significantly reduced IL-4 production within germinal centers (arrowheads indicate HuCD2^+^ CD4^+^ cells, Fig 3A). In addition to reduced frequency, immunofluorescence analysis revealed that germinal center area was reduced in the draining lymph node of mice born to infected mothers (Fig 3A). Subsequently, we analyzed TFH cells and observed a significant reduction of bulk TFH cells at 8 days post-immunization in offspring from infected mothers (Fig 3B), but no change in individual TFH cell production of IL-21, IFNγ, of IL-4 (Fig 3C and 3D). This TFH cell reduction was accompanied by reduced effector and memory Th2 responses (Fig 3E and 3F). Additionally, mice from *S*. *mansoni* infected mothers had significantly reduced numbers of germinal centers and IgG1^+^ B cells by flow cytometry (Fig 3G, 3H and 3J). We also observed a defect in the bulk memory B cell response in the pups from infected mothers (Fig 3I and 3K). This fact suggests that maternal infection suppresses the cellular immune response to heterologous antigens such as tetanus/diphtheria immunization.

### *S*. *mansoni* infection during pregnancy correlates with a weakened immune response in offspring at 14 days post-immunization

**Fig 4:**
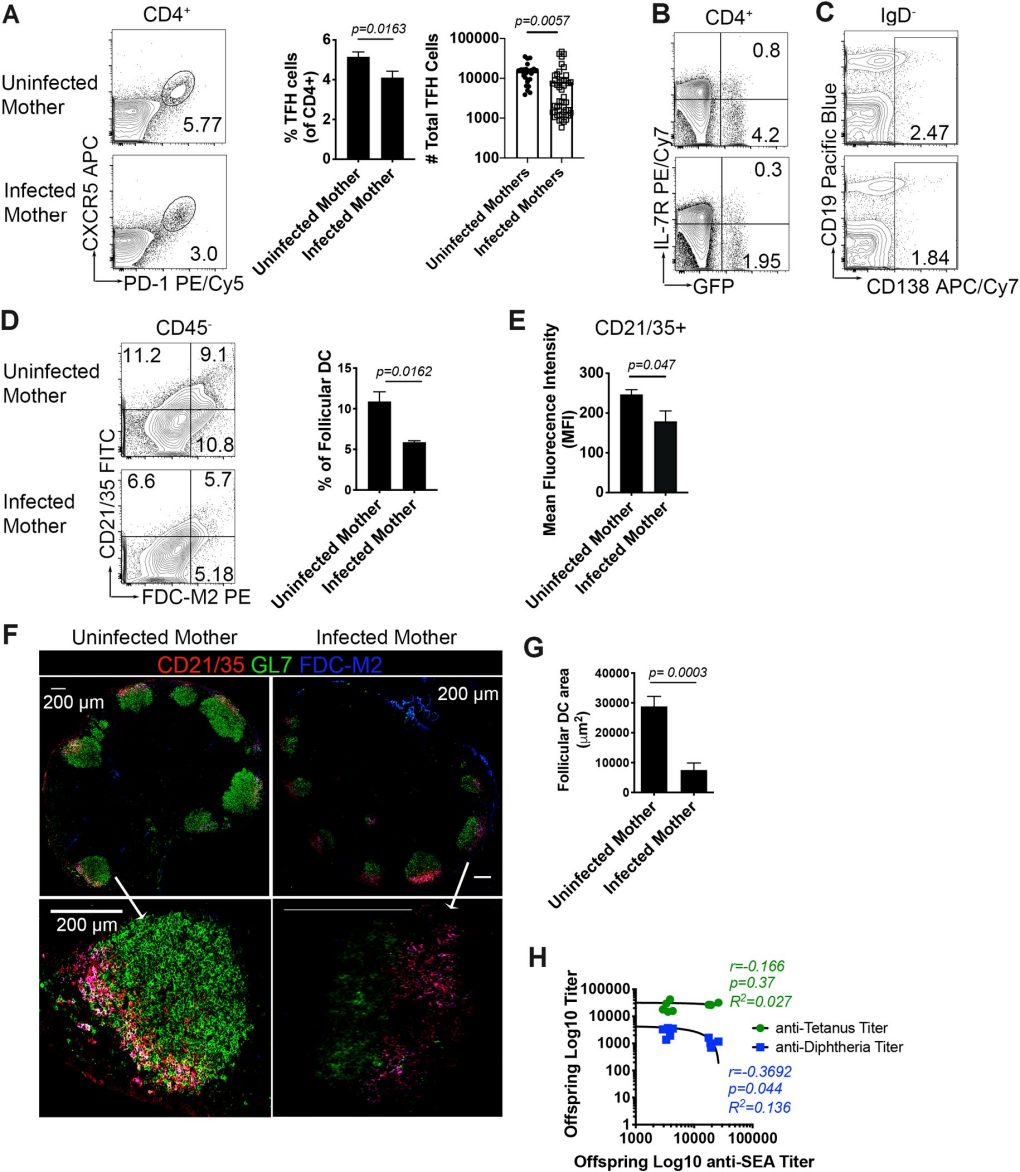
Pups from infected mothers exhibit reduced GC B cells, TFH, and FDC 14 days post immunization with Tetanus/Diphtheria. (A) TFH (PD-1^+^CXCR5^+^, gated from CD4^+^) flow plots, frequency, and cell number from draining PLN. (B) Th2 effector (GFP^+^IL-7R^-^) and Th2 memory (GFP^+^IL-7R^+^) from reactive lymph node (C) Plasma cells (IgD^-^CD19^+/-^CD138^+^) (D) FDC (CD21/35^+^FDC-M2^+^CD45^-^) frequencies (E) Median fluorescence intensity of CD21/35 on follicular dendritic cells. (F) Cryosections of reactive popliteal lymph node stained with CD21/35 (red), GL-7 (green), and FDC-M2 (blue). Scale bar: 200um. (G) Bar graph of the area of follicular dendritic cells the B cell follicles of draining lymph nodes. (H) Correlation analysis of offspring anti SEA titers to titers of anti-diphtheria and anti-tetanus 14 days post-immunization. Flow plots and confocal microscopy data are representative of three independent experiments with 3–6 mice per group. Statistical analysis was calculated with Student’s t-test. Correlation was expressed as Pearson correlation coefficient. Error bar denotes mean ± SEM.

In order to determine the effects of antenatal infection on the development of humoral response, we immunized 4get/KN2 pups with a commercial Tetanus/Diphtheria vaccine as above. At 14 days post-primary immunization, we observed a defect in TFH cells (Fig 4A) that corresponded with a reduced memory and effector Th2 response (Fig 4B) and decreased plasma cells in pups born to infected mothers (Fig 4C). Examining the FDC compartment, we found that maternal infection leads to decreased antigen-induced FDC expansion and CD21/35 production (quantified by flow cytometry Fig 4D and 4E). In addition to reduced frequency, pups from infected mothers exhibited reduced area and mean fluorescence intensity (MFI) of FDCs and germinal centers in draining peripheral lymph nodes (Fig 4F and 4G). We then asked if the observed reduction in bulk plasma cells and reduced germinal center area led to reduced anti-tetanus or anti-diphtheria titers. We found no correlation between offspring anti-SEA IgG1 titers and anti-tetanus titers, but we did find a correlation (R^2^ = 0.136, p = .044) between anti-SEA and anti-diphtheria IgG1 titers, suggesting that the diphtheria specific response is more adversely affected by antenatal schistosome exposure than the anti-tetanus response. This is likely tied to the reduction in IL-4 secretion in the germinal center reaction, as our recent work demonstrated that tetanus and diphtheria have a differential dependence on IL-4 for IgG1 class switching [21].

### Schistosomiasis during pregnancy alters the memory response to heterologous antigens in the offspring

**Fig 5:**
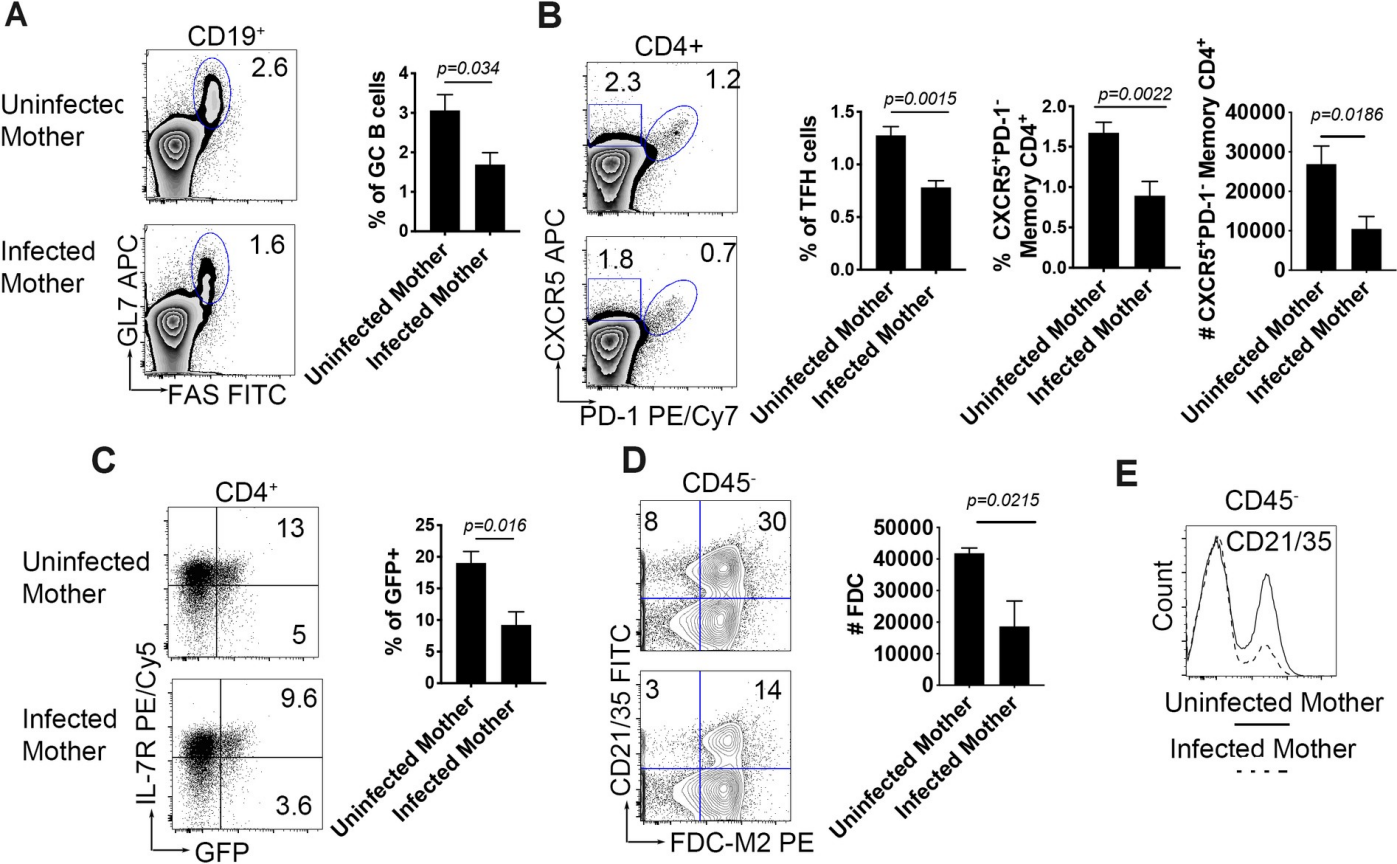
Cellular T and B responses are reduced in 4get/KN2 offspring from infected mothers >60 days post-primary immunization. 4get/KN2 28–35 days old were immunized in the hind footpad as described in the Materials and Methods. Draining popliteal lymph nodes were collected >60 days post primary immunization (A) GC B cells (CD19^+^GL-7^+^FAS^+^) frequencies (B) TFH(PD-1^+^CXCR5^+^, gated from CD4^+^) frequencies, (C) Th2 responses (GFP^+^IL-7R ^+/-^) (D) Flow cytometry analysis of CD21/35 cell count (E) FDC (CD21/35^+^FDC-M2^+^CD45^-^) frequency. Flow data is concatenated representative of n n>4 mice per group and two independent experiments. Statistical analysis was calculated with Student’s t-test.

Immunological memory is key to a protective immune response. Our previous work has demonstrated that subcutaneous immunization induces a persistent germinal center that continues to generate antigen-specific B cells at a low level, and that these memory B cells are critical for an accelerated secondary immune response [17]. Examination of the response to tetanus/diphtheria during maternal schistosomiasis revealed that at 60–90 days post immunization, mice born to infected mothers exhibited a defect in germinal center persistence (Fig 5A) accompanied by reduced TFH cells and the absolute number of memory TFH precursors (CXCR5^+^PD1^-^) (Fig 5B). The Th2 response (IL-4 transcription) was also significantly reduced in pups from infected mothers compared to their uninfected counterparts, with reductions in both effector (IL-7R^low^) and memory (IL-7R^hi^) Th2 compartments (Fig 5C). This correlated with a marked defect in CD21/35 expression in stromal (mesenchymal) cells (Fig 5D) and overall reduced frequency and number of FDCs in the draining lymph node (Fig 5D and 5E).

### Secondary immune responses to tetanus/diphtheria are compromised in offspring born to *S*. *mansoni* infected mothers

**Fig 6:**
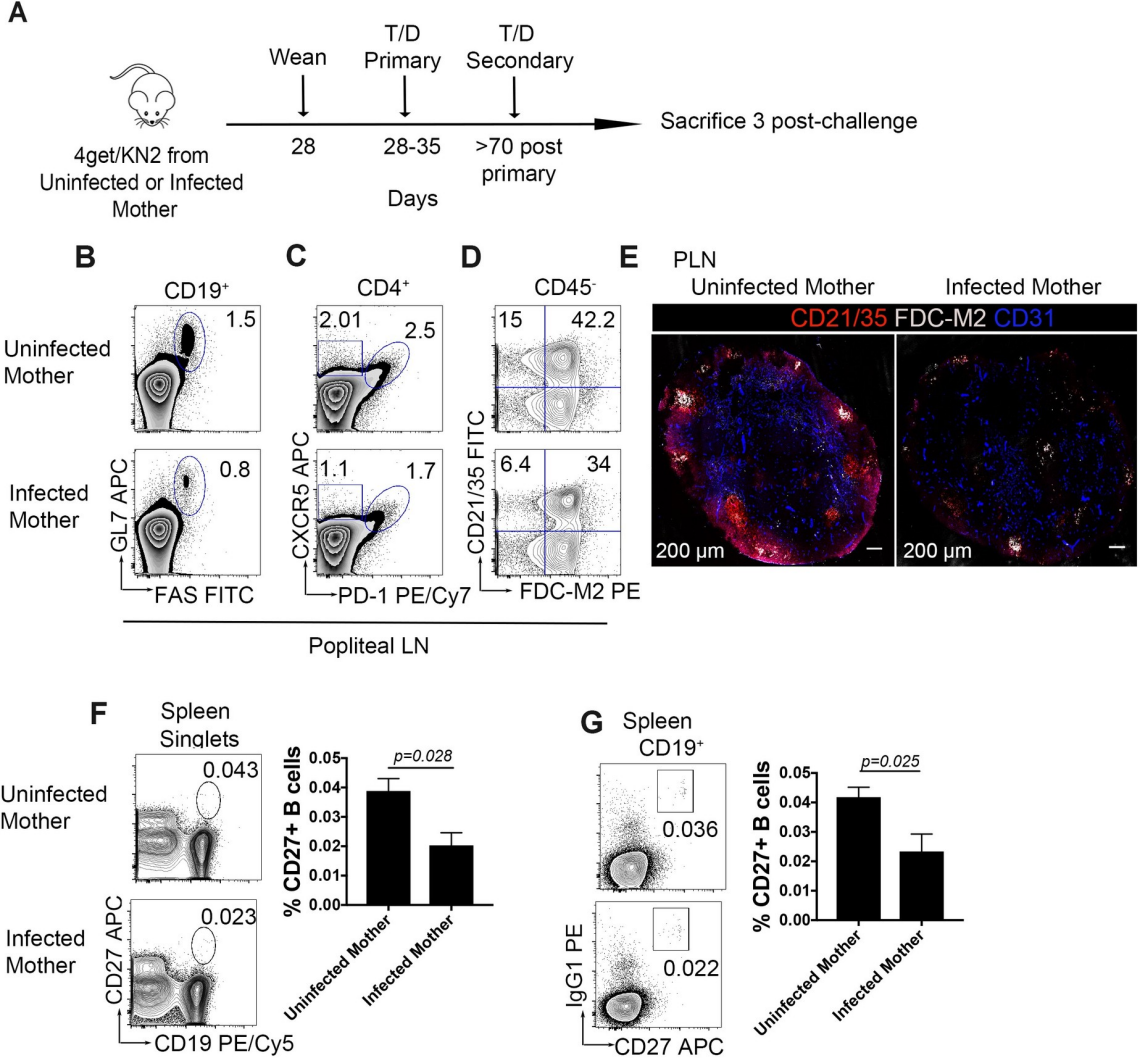
Memory B cell response is impaired during secondary challenge in offspring that were prenatally exposed to SEA antigens. (A) Schematic of experimental timeline (B) Flow cytometry of GC B cells (CD19+GL-7+FAS+), (C) TFH (PD-1+CXCR5+, gated from CD4+) (D) Follicular dendritic cells (CD21/35+FDCM2+ CD45-) (E) Tile confocal imaging of draining lymph node in response to secondary immunization with alum adjuvanted Tetanus/Diphtheria, cryosections were stained for CD21/35 (red), FDC-M2 (gray) and CD31 (blue). (F) Bulk memory B cells (CD19+CD27+) and (G) IgG1 memory response (IgG1+CD27+). Flow data are representative of n>4 mice per group and three independent experiments (except panel F and G, one independent experiment). Tile confocal imaging is representative of two independent experiments with n>3 mice per group per experiment.

In order to analyze the secondary response in maternal infection, pups were immunized for the first time at 28 to 35 days old. After 70–90 days-post primary immunization, pups were challenged with a second dose of adjuvanted Tetanus/Diphtheria vaccine subcutaneously (Fig 6A). In comparison to pups born to uninfected (control) mothers, pups from infected dams had reduced germinal center and TFH populations (Fig 6B and 6C). Moreover, FDCs were reduced approximately 40% in pups from infected mothers, suggesting a diminished antigen induced expansion. Consistent with previous timepoints, fluorescence intensity of complement receptor 1 and 2 (CD35 and CD21) were reduced in draining peripheral lymph nodes. This was accompanied by decreased fluorescent intensity of FDC-M2 (Fig 6E). Interestingly, the reduction in T and B cells during a recall response correlated to a significant reduction in bulk memory B cells (Fig 6F) and IgG1 memory B cells (Fig 6G) in the spleen, which highlights the relevance of the memory pool for cellular responses during a secondary response.

### Maternal schistosomiasis transcriptomically modulates the B cell lineage in an egg antigen-dependent manner

**Fig 7:**
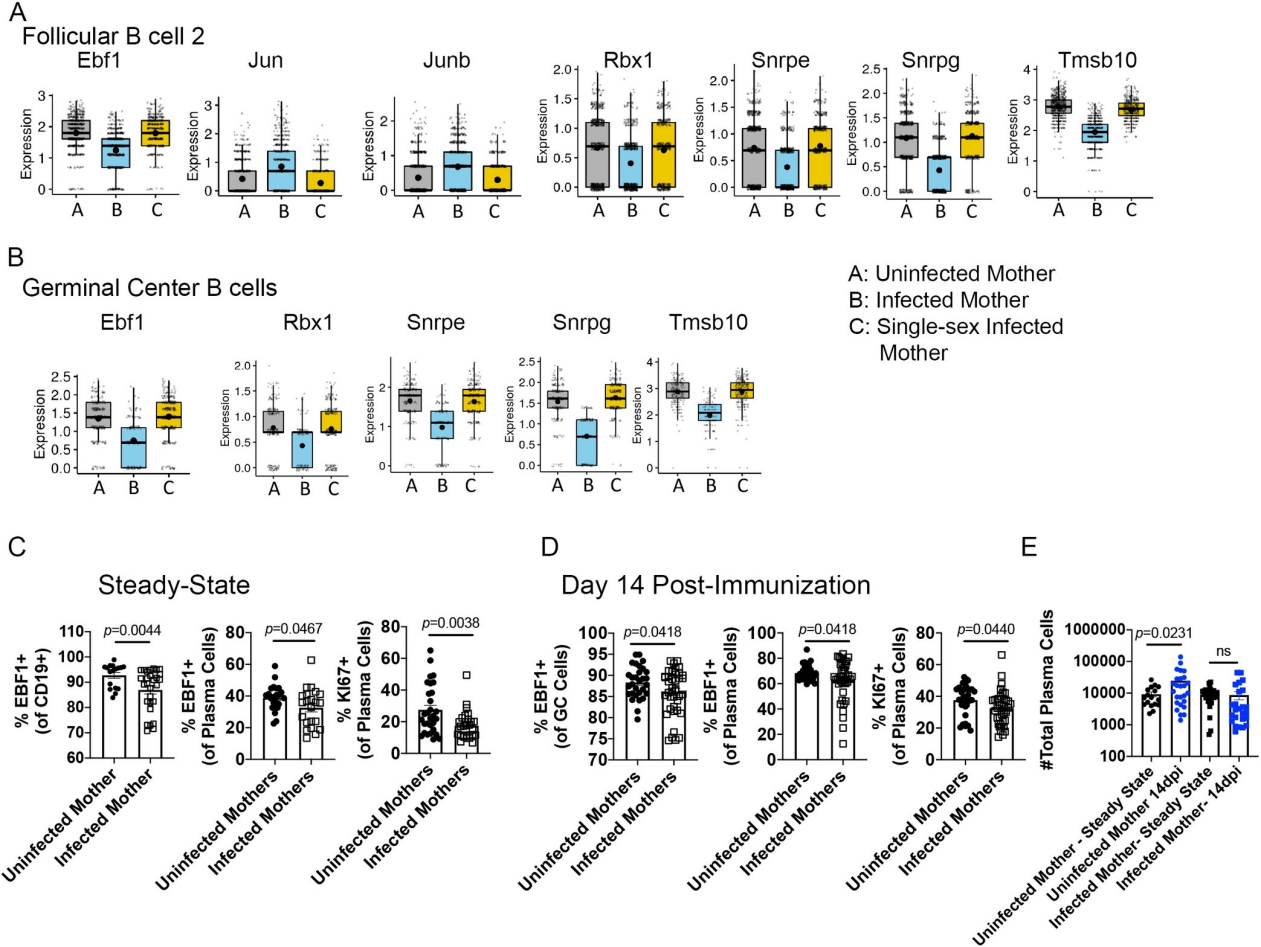
Maternal schistosomiasis induced modulation of B cell cell-cycle and proliferation is dependent on egg antigen exposure. (A, B) 4get homozygous females were infected with a low dose of *S*. *mansoni* cercariae to produce either single-sex infection (verified by anti-SEA ELISA and perfusion of the mother), or mixed-sex patent infection. Offspring born to mothers with either patent (egg exposure), single-sex (adult antigen exposure only), or control uninfected mothers were immunized at 28–35 days of age with commercial alum adjuvanted Tetanus/Diphtheria. At 8 days post-immunization, CD45^+^live cells were sorted and processed for 10x Genomics single-cell sequencing. Expression levels of genes significantly altered in follicular B cluster 2 and germinal B cell clusters. In these plots, each dot represents a single cell. Normalized expression values were used, and random noise was added to show the distribution of data points. The box plots show interquartile range and the median value (bold horizontal bar). The average expression value per sample is indicated by the dot. Wilcoxon’s test was used for statistical comparisons. (C) Flow cytometry analysis of Ebf-1 and Ki-67 at steady state in CD19^+^ and plasma B cells. (D) Flow cytometry analysis of Ebf-1 and Ki-67 at day 14 post tetanus/diphtheria immunization. (E) Total number of plasma cells in the popliteal lymph node at steady-state and Day 14 post-tetanus/diphtheria immunization. Statistical analysis for C-E was calculated with Student’s t-test with Welch’s correction.

In order to more fully understand the molecular effects of maternal schistosomiasis, we performed single-cell RNASeq (scRNASeq) using the 10x Genomics platform. Offspring from infected, uninfected, and single-sex infected mothers (verified by anti-SEA ELISA and perfusion of the mother) at 10–12 weeks post infection (6–8 offspring per group) were immunized with tetanus/diphtheria at 28 days of age. On day 8, post-immunization live CD45^+^ immune cells were sorted from the pooled draining lymph nodes of each group. We aggregated data from 13,437 individual cells (4,879 cells from uninfected mothers; 4,388 cells from infected mothers; 4,170 cells from single-sex infected mother) and performed unsupervised clustering analysis based on the similarity of gene expression signatures by using the Seurat single-cell genomics R package [22]. This analysis revealed 23 distinct cell clusters representative of both lymphoid and myeloid lineages, and cluster identity was determined using CIPR [23]. For cell clusters in which the algorithm could not make a clear call such as TFH cells, we resorted to differential expression analysis between clusters to identify distinguishing markers (S2A Fig). Patent maternal infection (Group B) shifted the cellular proportions of the CD45^+^ pool, reducing germinal center B and TFH cells while increasing CD8^+^ and naive CD4^+^ cells (S2B Fig). The cellular proportions in offspring born to single-sex infected mothers is closer to that of uninfected mothers than patent infection. Upon confidently identifying cell clusters, we analyzed the differences between offspring exposed to egg antigens via patent infection and those unexposed or exposed only to adult antigens (single-sex infected, group C). Since our other experiments found a profound defect in the B cells response to immunization, we focused on the follicular and germinal center clusters. Offspring born to patently infected mothers had a shared modulation of the key B cell lineage transcription factor Ebf-1, as well as multiple genes involved in cell-cycle/proliferation. The follicular B cell clusters shared a similar transcriptional profile (Figs 7A and S3 Fig), with follicular B cell cluster 2 having a significant reduction in Ebf-1 (adj p = 1.83e-59), a significant increase in Jun (adj p = 2.89e-18) and Junb (adj p = 3.92e-15), and significant decreases in Rbx-1 (adj p = 1.68e-13), Snrpe (adj p = 1.75e-26), Snrpg (adj p = 5.75e-83) and Tmsb10 (adj p = 3.55e-149). The germinal center B cell cluster had reduced expression of Ebf-1 (adj p = 7.05e-14), and significant decreases in Snrpe (adj p = 3.27e-20), Snrpg (adj p = 4.57e-30), Rbx1 (adj p = 8.15e-7), and Tmsb10 (adj p = 6.95e-32). Importantly, offspring born to single-sex infected mothers have no significant alterations in these genes in any B cell cluster, indicating that transcriptional alteration of cell cycle and B cell identity is dependent on egg antigen exposure, which supports our data correlating maternal anti-SEA titer to offspring anti-SEA titer, and offspring anti-SEA titer to offspring anti-diphtheria titer. We were unable to identify a distinct plasma cell cluster in the single-cell data, so we measured EBf-1 and cell cycle via flow cytometry. At steady state (28–35 days of age), offspring born to patently infected mothers have reduced Ebf-1 expression in plasma cells (Fig 7C). Ki67 is a widely-used marker of active proliferation [24] so we quantified Ki-67 expression in steady-state and found that a significantly lower percentage of plasma cells from offspring born to infected mother are Ki-67^+^ (Fig 7C), suggesting reduced proliferation or cell cycle progression in these cells. We then measured Ebf-1 and Ki-67 at day 14 post-immunization (the peak of the lymph node B cell response) and found that both germinal center and plasma cells in offspring born to infected mothers have reduced EBF-1 (Fig 7D), and plasma cells have reduced Ki-67 (Fig 7D). Indeed, when we measure the absolute number of plasma cells at steady-state and day 14 post immunization we find that lymph node plasma cells in offspring from uninfected mother expand, but the offspring from infected mother fail to expand the plasma cell compartment in response to tetanus and diphtheria antigens (Fig 7E). These data strongly support the conclusion that *in utero* exposure to schistosome egg antigens modulates the cell cycle and proliferative capacity of B cells, resulting in deficient plasma cell production to both microbiota and immunization.

## Discussion

In this study, we report the generation of a maternal *Schistosoma mansoni* infection model in dual IL-4 reporter mice. Our results indicate that *in utero* egg antigen sensitization occurs, which is consistent to what has been published by other authors [25] and confers a state of immune-hyporesponsiveness in the offspring at steady state, this study does not differentiate between trans-placental antigen exposure and sensitization through breastfeeding; however, it has been previously suggested that exposure to parasitic antigens, either *in utero* or via breast milk, diminishes the heterologous response [25], and in some other cases nursing by infected mothers protected offspring against infection with the same helminth [26]. We observed anti- *S*. *mansoni* egg antigen-specific IgG1 (the dominant isotype produced against egg antigens [17]) at 35, 90 and 180 days of age in mice from infected mothers and no detectable titers in mice born to uninfected controls, suggesting either maternal antibody, antibody-secreting cell transfer to the offspring, or *in situ* antibody production by the offspring. The placental transfer of different IgG subclasses has been well documented [27–29]. In our model, we observed maternal antibodies in KN2 homozygous (IL-4 deficient) pups at 35, 60, and 90 days of age, suggesting a sustained antibody response to SEA from either maternal antibodies or cells, since our previous work has demonstrated that IL-4 deficient mice fail to mount an IgG1 response to SEA [17]. The presence of an antigen-specific humoral response can potentially translate to protection, or alternatively, inhibit vaccination-induced protection, as is the case for maternal antibodies to measles and other vaccinations, in humans and other mammals [30]. In humans, the persistence of maternally-derived antibodies is approximately 6–12 months, while in other mammals, it varies from 3 to 6 months, depending on the species. [31]. Nevertheless, offspring born to infected mothers have a circulating humoral response specific to *S*. *mansoni*; future work will determine what proportion of these cells are of maternal versus fetal origin. Future work will also focus on elucidating the contribution of maternal antibodies in the offspring’s response to tetanus and diphtheria. Previous reports have documented the presence of profibrotic biomarkers (such as desmosin, connective tissue growth factor (CTGF), Matrix Metallopeptidase 2 (MMP-2), among others) in cord blood from neonates born to mothers infected with *S*. *japonicum*, which was attributed to the fetal response to schistosome antigens, as the transfer of collagen markers was unlikely, given that active transplacental transfer of collagen markers has not been observed in humans [32].

Interestingly, our assessment of the immune response of offspring at steady-state revealed that bulk antibody responses were impaired in offspring from infected mothers in comparison to their uninfected counterparts. This state correlated with diminished FDC populations in the peripheral lymph nodes. In addition to this, the area of complement receptor 1 and 2 positive FDCs in B cell follicles is also reduced at steady state, and BaffR expression on B cells is reduced. Collectively, these findings are consistent with a role for FDCs in the impaired maintenance of plasma and memory B cells seen in offspring from infected mothers. FDCs are the stromal cells located in the germinal center that retain antigen for a long period and bind B cells to prevent apoptosis. These cells have previously been demonstrated to be fundamental for maintenance of plasma cells and long-lived memory B cells [33]. Recent work in our laboratory has demonstrated that IL-4 is required for FDC maintenance and positioning at steady-state via induction of lymphotoxin α/β [21]. In light of that, we sought to determine the cellular source of steady-state lymph node IL-4 production in this model. We found that iNKT cells are the major producers of IL-4 in control offspring during early life and that steady-state secretion of IL-4 is almost eliminated in offspring born to infected mothers. This data suggests that the observed defects in FDC numbers and stimulatory capacity may be due to insufficient IL-4 during lymph node development and maturation. Bolstering this notion is the finding that there is a significant reduction in IL-4 competent (GFP^+^) CD4 T cells in the peripheral blood of these mice. This evidence suggests that prenatal helminth sensitization has a lasting effect on offspring immunity, and that offspring from mothers that were infected during pregnancy could be at a higher risk of infection with various pathogens due to a defect in homeostatic immunity as has been previously postulated [34], which will be the focus of future studies in our lab.

We further evaluated the immune response following primary immunization with a commercially available alum adjuvanted Tetanus/Diphtheria vaccine used in clinical settings. There is extensive evidence in humans of impaired response to immunization [7,35], but little is known of the mechanism(s) controlling this defect. We observed that following primary immunization, the pups from infected mothers exhibit a defect not only in the expansion of FDCs, but also in the development of T follicular helper and germinal center B cells. We hypothesize that the combined FDC and TFH deficiency leads to the diminished response to primary immunization, as well as diminished memory T and B cell responses. Indeed, at 8-days post-immunization, there is a reduction of bulk memory B cells and IgG1^+^ B cells, which could potentially have an impact on the long-term maintenance of humoral immunity necessary for protection following vaccination. This observation is bolstered by our data at day 14 post-immunization where germinal center formation in a reactive lymph node was impaired in 4get/KN2 pups from infected mothers in comparison to pups from uninfected mothers. Acute antigen exposure causes lymphocyte proliferation, which is followed by a pool of quiescent long-lived IL-7R^+^ memory cells capable of potent response to challenge with the same antigen. [36]. We have previously determined that in a type 2 response, secondary TFH cells are generated in large part by recruitment of circulating Th2 memory T cells (IL-7R^+^GFP^+^) back to the lymph node, and that their interaction with memory B cells is critical to the secondary plasma cell response [17]. In our model of maternal schistosomiasis, we observed a diminished pool of both populations of IL-7R^+^GFP^+^CD4^+^ (memory Th2 cells) and IL-7R^-^GFP^+^CD4^+^ cells (Th2 effector cells) and TFH circulating precursors (CXCR5^+^PD-1^-^CD4^+^). This was accompanied by a reduction in the frequencies of TFH cells and plasma cells, as well as a diminished secretion of IL-4 cytokine in the germinal center of the reactive lymph node (visualized in confocal tile scans). In addition to the defects observed in T cells, we also observed reduced FDC and bulk memory B cell population. Overall, this indicates that signals required for mounting a robust adaptive immune response to vaccination were dampened following prenatal exposure to *S*. *mansoni* antigens. To identify potential molecular mechanisms underlying the observed B cell dysfunction, we performed scRNASeq on offspring from uninfected, patently infected, and single-sex infected mothers on the 10x Genomics platform. Parasites of the genus Schistosoma are dioecious and need both sexes to produce a patent infection with egg production. Therefore, single-sex Schistosoma infections allow the dissection of the contributions from adult worm antigens versus the egg antigen that drives immunopathology in the host, and the development of chronic regulatory and Th2 responses. Thus, single-sex infections allow for a controlled setting of schistosome infection life cycle components. Analysis of the cluster level response revealed a coordinated modulation of follicular and germinal center B cells, with a profound reduction in the lineage defining transcription factor Ebf-1, and multiple genes involved in cell cycle/proliferation, such as Snpre and Snprg, which are part of the spliceosome machinery whose overexpression has been shown to drive cell proliferation in multiple models [37,38]; Rbx1, a known modulator of DNA replication licensing proteins [39]; Tmsb10, a regulator of actin cytoskeleton with roles in B cells [40,41]. Concomitantly, Jun and Junb, which are involved in both B cell differentiation and B cell receptor signaling [42,43] were increased in these clusters. We validated that Ebf-1 expression is reduced in both bulk CD19^+^ and plasma cells at steady state (28–35 days of age) and found that a significantly lower proportion of steady-state plasma cells express Ki-67, a state that persists following immunization, strongly indicating that there is indeed a defect in B cell expansion and differentiation in response to foreign antigens.

Coinciding with defects in B cell proliferation, we find a consistent defect in follicular dendritic cell expansion and expression of CD21/35. While our data suggest a role for iNKT cell IL-4 production in FDC maintenance, the data is not definitive. Future work will focus on identifying the specific homeostatic role of iNKT cell-derived IL-4, and the molecular mechanism that underlies both iNKT cell production of IL-4, and diminished FDC cell function in the context of maternal infection. There is a body of literature supporting the many effects of the prenatal environment on inflammatory diseases during adolescence and adulthood. It has been hypothesized that inflammatory responses and infections during pregnancy might alter epigenetic profiles in the fetus [44]. Future studies linking the epigenetic effects of schistosomiasis infection during pregnancy and immune responses in offspring will help elucidate potential pathways involved in the immune hypo-responsiveness observed in this model.

One of the key goals of vaccination is to induce an immunological memory that is protective upon re-challenge with the same antigen [45–47]. Hence, we explored whether there was a difference between pups from infected and control mothers in the development of a sustained germinal center and maintenance of memory B and T cell pools. Pups were immunized, and their immunological responses were assessed at >60 days post immunization. Surprisingly, offspring from infected mothers mounted a weaker cellular response, with significantly smaller germinal centers that correlated to a significant reduction in long-lived TFH, memory Th2 cell, and FDCs, as well as reduced complement receptor 2/1 (CD21/35) expression. Importantly, our previous work [17] has established precursor bulk memory T cells (CXCR5^+^PD-1^-^) as critical to the secondary TFH cell response, and this cell population was significantly reduced at the memory timepoints in pups from infected mothers in comparison to pups from uninfected mothers. Importantly, both memory T and B cell responses remained defective following secondary immunization with tetanus/diphtheria, indicating that these cellular defects are long-lived. These data strongly support the premise that *in utero* and nursing exposure to schistosome egg antigens transcriptionally reprograms the proliferative potential of the B cell lineage to foreign antigens, as well as the immune complex presenting capacity of FDCs, leading to long-lived suppression of the stromal-lymphocyte axis and the ability to mount a B cell response to immunization. Previous work has demonstrated differential effects of schistosome antigen exposure between *in utero* and nursing [48], future work will determine the role of these different exposures on IL-4 production and the stromal/lymphocyte axis. There is heterogeneity in the epidemiological data about maternal helminth infections, with maternal schistosomiasis correlating with reduced humoral immunity to only some childhood vaccines. Future studies elucidating whether a similar mechanism impacts the responses to other heterologous antigens will be necessary to broaden our understanding of maternal infection in offspring health.

## Materials and methods

### Ethics statement

Experimental procedures involving mice were approved by the Institutional Animal Care and Use Committees of the University of Utah (18–09001) and Purdue University (1406001081A001).

### Mice strains and *in vivo* treatments

4get homozygous (Il4^tm1Lky^, The Jackson Laboratory), KN2 homozygous (Il4^tm1(CD2)Mmrs^, a gift from Markus Morhs [16]) and 4get/KN2 were bred in-house under specific pathogen-free (SPF) conditions at Purdue University and the University of Utah. Homozygous 4get or heterozygous 4get/KN2 females of 7–8 weeks of age were either infected with a low dose (35 cercariae) of *Schistosoma mansoni* or mock-infected. Infection was performed by exposing mice percutaneously to the parasite cercariae for 25 minutes. Infected and uninfected females (as controls) were bred with naive KN2 homozygous male mice at five-six weeks post-infection and resulting 4get/KN2 pups were weaned and genotyped (as necessary) at 28 days old (S1 Fig). Females typically plugged within 1 week of pairing. Infected and control females were kept for the production of multiple litters, with each female generating 3–6 litters. A mix of age-matched female and male pups either from control or infected females were sacrificed at 28-35-days old for steady-state studies. Other experimental mice were immunized with 1/10^th^ of the human dose of Tetanus/Diphtheria commercial vaccine (Tetanus and Diphtheria Toxoids Adsorbed, MassBiologics, Boston, Massachusetts) subcutaneous (s.c.) in the rear footpad and mice were sacrificed at 8, 14, and over 60 (memory) days post-immunization. Secondary immunizations were performed at over 60 days post primary immunization, and mice were sacrificed 3 days post-secondary immunization. Data presented throughout the manuscript are from litters born to mothers from 9–26 weeks post-infection.

### Isolation of cells and flow cytometry

Cells were isolated from popliteal lymph node (pLN), hepatic lymph node (hLN), whole blood, and spleen. For follicular dendritic cells (FDC) analysis, tissues were digested as previously described [21,49]. Briefly, lymph nodes were digested at 37°C for 20 minutes, with occasional inversion of the contents and filtered through 100 μm filters. Single-cell suspensions were stained with surface markers, and intracellular staining was performed as previously described [50]. Antibodies conjugated with the following fluorochromes were used: allophycocyanin, allophycocyanin-Cy7, Pacific blue, Brilliant Violet 510, Brilliant Violet 605, Brilliant Violet 650, Brilliant Violet 711, Brilliant Violet 786, Super Bright 600, FITC, Phycoerythrin, PE/Cy7, PE/Cy5, efluor660, PerCpCy5.5, APCAlexafluor700, and Zombie Red. The following antibodies from eBioscience, BD Biosciences, Biolegend were used: CD3 (17A2), CD4 (RM4-5), CD19 (1D3), CD138, (281–2), IgG1 (A85-1), IgD (11–26), GL7 (GL7), CD45 (30-F11), CD31 (390), podoplanin (eBIO 8.1.1), FDC-M2 (FDC-M2), CD21/35 (7G6), PD-1 (J43), CXCR5 (2G8), TCRβ (H57-597), DX5 (CD49b), CD27 (LG.3A10), IFNγ (XMG1.2), IL-7R (SB1199), FAS (15A7), HuCD2 (RPA-2.10), Ki-67 (sola15), Ebf-1 (T26-818), BaffR (7H22-E16), CD38 (90), and IL-21R/FC chimera (R&D Systems; 596-MR-100). Tetramer staining was performed using Mouse CD1d-tetramer loaded with the α-GalCer analog PBS57, and unloaded mouse CD1d-tetramer was used as staining control. Both loaded and unloaded tetramers were provided by the NIH Tetramer Core Facility.

### Immunofluorescence microscopy

Whole tissue (popliteal lymph node or hepatic lymph node) was collected and placed in Tissue-Tek optimum cutting temperature compound (OCT) (Thermo Scientific) and frozen in liquid nitrogen. Serial cryostat sections (10μm) were collected using a Leica CM 1850. Sections were then air-dried and fixed in ice-cold 75% acetone/25% ethanol for 5 mins. Sections were rehydrated in PBS for 5 to 10 minutes and blocked using a biotin blocking kit (Vector Laboratories) followed by incubation with 1%(v/v) in PBS of rat and rabbit serum. Staining with appropriate antibodies was done overnight at 4°C followed by secondary staining for 1 hour at room temperature. Coverslips were mounted using ProLong anti-fade reagents (Life Technologies). Images were acquired with a Leica TCS Sp5 Laser Scanning Microscopy with an average grid size of 3x3. Images were taken with a 20x objective at a resolution of 1024x1024. Image post-processing was done using Fiji is Just ImageJ software (1.47v).

### ELISA

Schistosoma mansoni egg antigens (SEA) and tetanus and diphtheria-specific IgG1 endpoint titers were determined by enzyme-Linked immunosorbent assay (ELISA) using the mAb X56 (BD) and Immulon 4HB plates (Thermo Fisher Scientific) as previously described [21]. Briefly, plates were coated with 2μg/mL tetanus (List Labs), diphtheria (Sigma), or SEA. The following morning plates were blocked with 1% milk and incubated with serial dilutions of primary antibody, followed by incubation with anti-mouse IgG1 ads-HRP antibody (Southern Biotech) and ABTS substrate. Plates were read at 405 nm at room temperature on a BioTek plate reader.

### Single Cell RNASeq

#### Sample preparation and sequencing

Single-cell suspension form 6–8 draining lymph nodes per group were pooled and stained with Dapi (Sigma) and anti-CD45. Live CD45+ cells were sorted via BD FACSAria cell sorter and washed once in PBS containing 0.04% BSA. Samples were then processed for SCseq via a 10× platform according to the manufacturer’s instructions (10× Genomics). Paired-end RNASeq (125 cycles) was performed via an Agilent HiSeq next-generation sequencer. Sequencing reads were processed by using a 10x Genomics CellRanger pipeline and further analyzed with the Seurat R package. The effect of mitochondrial gene representation and the variance of unique molecular identifier (UMI) counts were regressed out from the data set prior to analysis. Gene expression signatures defining cell clusters were analyzed after aggregating 3 samples (uninfected mother, infected mother, single-sex infected mother). The raw data from scRNASeq experiments in this manuscript can be found in the NCBI’s Gene Expression Omnibus database (GSE162075).

### Identification of cell clusters

Cells in our data set were clustered by using the FindClusters function of the Seurat analysis package, which identifies clusters via a shared nearest neighbor (SNN) modularity optimization-based algorithm. This function identified 23 distinct clusters spanning the lymphoid and myeloid cell lineages. The biological identities of cell clusters were annotated with the help of an immune-cell scoring algorithm ([23] and available at http://labs.path.utah.edu/oconnell/resources.htm) and by surveying known immune cell markers in the SCseq data set. The immune-scoring algorithm compares the gene signatures of the cell clusters in this study with the publicly available microarray data hosted in the Immunological Genome Project Database (ImmGen). By using differentially expressed gene signatures from Seurat, the immune-scoring algorithm performs the following steps: (a) for each ImmGen cell population, and each gene found in ImmGen microarrays, it calculates the ratio of normalized microarray signal to the average signal value of the gene from the whole ImmGen data; (b) applies natural log transformation to the ratio, resulting in positive numbers for upregulated genes and negative numbers for downregulated genes in ImmGen data sets; (c) multiplies ImmGen log-ratio values with the log-ratio of matching genes that are differentially expressed in each cell cluster in the SCseq dataset; (d) sums up scores from all the genes to yield an aggregate identity score for each ImmGen cell type for a given SCseq cluster. In this approach, genes that are differentially upregulated or downregulated in both ImmGen and SCseq data sets contribute to the immune identity score more heavily (a positive number is obtained when 2 log-ratio values with the same sign are multiplied). In contrast, if a gene is inversely regulated in ImmGen and SCseq clusters, the immune identity score is reduced. Through this method, the correlation between the gene expression signatures of SCseq cell clusters in our study and ImmGen data subsets assists in determining the cluster identities. In cases where this algorithm is unable to make a clear call (as T follicular helper cells and myeloid cells), we surveyed the expression of known genes in the data set and performed differential expression analyses between closely related cell clusters. This approach allowed us to differentiate the subsets and T cell clusters further. Upon naming the clusters, the Seurat R package was used to create plots for the expression of selected genes. GSEA analysis was performed by using fgsea R package [51], after ranking genes using a signal-to-noise metric [52].

### Statistical analysis

Statistical analyses were performed using either a non-parametric Mann-Whitney test, unpaired Student’s t-test, or ANOVA with Bonferroni’s multiple comparison test based on the distribution of the data. p values ≤ 0.05 were considered statistically significant. To test the strength of the association between variables, Pearson correlation coefficient was calculated. Graph generation and statistical analyses were performed using Prism (GraphPad v8.0).

## Supporting information

S1 Fig Schematic of Experimental Maternal Schistosomiasis setup. (TIF)

S2 Fig A) scRNAseq UMAP plots showing the immune landscape in showing the immune landscape in the draining lymph node in offspring born to uninfected, infected, and single-sex infected mothers at day 8 post tetanus/diphtheria immunization. B) Frequency of cell clusters with the indicated identity in offspring born to uninfected, infected, and single-sex infected mothers.
(TIF)

S3 Fig Expression levels of Cell cycle and B cell genes within the Follicular B cells are shown. In these plots, each dot represents a single cell. Normalized expression values were used, and random noise was added to show the distribution of data points. The box plots show interquartile range and the median value (bold horizontal bar). Average expression value per sample is indicated by the center black points. Wilcoxon’s test was used for statistical comparisons. The listed values are adjusted p-values.
(TIF)

## References

[1] Olveda DU, Olveda RM, McManus DP, Cai P, Chau TN, Lam AK, et al The chronic enteropathogenic disease schistosomiasis. Int J Infect Dis. 2014;28:193–203. Epub 2014/09/25. 10.1016/j.ijid.2014.07.009 . 25250908

[2] Steinmann P, Keiser J, Bos R, Tanner M, Utzinger J. Schistosomiasis and water resources development: systematic review, meta-analysis, and estimates of people at risk. Lancet Infect Dis 2006;6(7):411–25. Epub 2006/06/23. 10.1016/S1473-3099(06)70521-7 . 16790382

[3] Colley DG, Bustinduy AL, Secor WE, King CH. Human schistosomiasis. Lancet 2014;383(9936):2253–64. Epub 2014/04/05. 10.1016/S0140-6736(13)61949-2 24698483 PMC4672382

[4] McDonald EA, Pond-Tor S, Jarilla B, Sagliba MJ, Gonzal A, Amoylen AJ, et al Schistosomiasis japonica during pregnancy is associated with elevated endotoxin levels in maternal and placental compartments. The Journal of infectious diseases. 2014;209(3):468–72. 10.1093/infdis/jit446 23964108 PMC3883168

[5] Siegrist D, Siegrist-Obimpeh P. Schistosoma haematobium infection in pregnancy. Acta Trop 1992;50(4):317–21. Epub 1992/04/01. 10.1016/0001-706x(92)90066-7 . 1356302

[6] Kurtis JD, Higashi A, Wu HW, Gundogan F, McDonald EA, Sharma S, et al Maternal Schistosomiasis japonica is associated with maternal, placental, and fetal inflammation. Infection and immunity. 2011;79(3):1254–61. 10.1128/IAI.01072-10 21149589 PMC3067505

[7] Ondigo BN, Muok EMO, Oguso JK, Njenga SM, Kanyi HM, Ndombi EM, et al Impact of Mothers' Schistosomiasis Status During Gestation on Children's IgG Antibody Responses to Routine Vaccines 2 Years Later and Anti-Schistosome and Anti-Malarial Responses by Neonates in Western Kenya. Front Immunol. 2018;9:1402 Epub 2018/07/04. 10.3389/fimmu.2018.01402 29967622 PMC6015899

[8] Malhotra I, Mungai P, Wamachi A, Kioko J, Ouma JH, Kazura JW, et al Helminth- and Bacillus Calmette-Guerin-induced immunity in children sensitized in utero to filariasis and schistosomiasis. J Immunol 1999;162(11):6843–8. . 10352306

[9] Elias D, Akuffo H, Pawlowski A, Haile M, Schon T, Britton S. Schistosoma mansoni infection reduces the protective efficacy of BCG vaccination against virulent Mycobacterium tuberculosis. Vaccine 2005;23(11):1326–34. Epub 2005/01/22. 10.1016/j.vaccine.2004.09.038 . 15661380

[10] Attallah AM, Abbas AT, Dessouky MI, El-emshaty HM, Elsheikha HM. Susceptibility of neonate mice born to Schistosoma mansoni-infected and noninfected mothers to subsequent S. mansoni infection. Parasitol Res 2006;99(2):137–45. 10.1007/s00436-006-0127-x . 16521039

[11] Santos P, Sales IR, Schirato GV, Costa VM, Albuquerque MC, Souza VM, et al Influence of maternal schistosomiasis on the immunity of adult offspring mice. Parasitology research. 2010;107(1):95–102. 10.1007/s00436-010-1839-5 . 20372927

[12] Rueff-Juy D, Faure M, Drapier AM, Cazenave PA. Role of maternal Ig in the induction of C kappa-specific CD8+ T cell tolerance. J Immunol 1998;161(2):721–8. Epub 1998/07/22. . 9670948

[13] Desowitz RS, Elm J, Alpers MP. Plasmodium falciparum-specific immunoglobulin G (IgG), IgM, and IgE antibodies in paired maternal-cord sera from east Sepik Province, Papua New Guinea Infect Immun 1993;61(3):988–93. Epub 1993/03/01. 10.1128/IAI.61.3.988-993.1993 8432619 PMC302830

[14] Friedman JF, Mital P, Kanzaria HK, Olds GR, Kurtis JD. Schistosomiasis and pregnancy. Trends Parasitol 2007;23(4):159–64. Epub 2007/03/06. 10.1016/j.pt.2007.02.006 . 17336160

[15] Mohrs M, Shinkai K, Mohrs K, Locksley RM. Analysis of type 2 immunity in vivo with a bicistronic IL-4 reporter. Immunity 2001;15(2):303–11. Epub 2001/08/25. 10.1016/s1074-7613(01)00186-8 . 11520464

[16] Mohrs K, Wakil AE, Killeen N, Locksley RM, Mohrs M. A two-step process for cytokine production revealed by IL-4 dual-reporter mice. Immunity. 2005;23(4):419–29. Epub 2005/10/18. 10.1016/j.immuni.2005.09.006 16226507 PMC2826320

[17] Fairfax KC, Everts B, Amiel E, Smith AM, Schramm G, Haas H, et al IL-4-secreting secondary T follicular helper (Tfh) cells arise from memory T cells, not persisting Tfh cells, through a B cell-dependent mechanism. J Immunol. 2015;194(7):2999–3010. Epub 2015/02/26. 10.4049/jimmunol.1401225 25712216 PMC4495582

[18] Tew JG, Wu J, Fakher M, Szakal AK, Qin D. Follicular dendritic cells: beyond the necessity of T-cell help. Trends Immunol 2001;22(7):361–7. Epub 2001/06/29. 10.1016/s1471-4906(01)01942-1 . 11429319

[19] Suzuki K, Grigorova I, Phan TG, Kelly LM, Cyster JG. Visualizing B cell capture of cognate antigen from follicular dendritic cells. J Exp Med 2009;206(7):1485–93. 10.1084/jem.20090209 19506051 PMC2715076

[20] Wu Y, Sukumar S, El Shikh ME, Best AM, Szakal AK, Tew JG. Immune complex-bearing follicular dendritic cells deliver a late antigenic signal that promotes somatic hypermutation. J Immunol 2008;180(1):281–90. Epub 2007/12/22. 10.4049/jimmunol.180.1.281 . 18097029

[21] Cortes-Selva D, Ready A, Gibbs L, Rajwa B, Fairfax KC. IL-4 promotes stromal cell expansion and is critical for development of a type-2, but not a type 1 immune response. Eur J Immunol 2019;49(3):428–42. Epub 2018/12/24. 10.1002/eji.201847789 . 30575951 PMC6953475

[22] Butler A, Hoffman P, Smibert P, Papalexi E, Satija R. Integrating single-cell transcriptomic data across different conditions, technologies, and species. Nat Biotechnol 2018;36(5):411–20. Epub 2018/04/03. 10.1038/nbt.4096 29608179 PMC6700744

[23] Ekiz HA, Conley CJ, Stephens WZ, O'Connell RM. CIPR: a web-based R/shiny app and R package to annotate cell clusters in single cell RNA sequencing experiments. BMC Bioinformatics 2020;21(1):191 Epub 2020/05/18. 10.1186/s12859-020-3538-2 32414321 PMC7227235

[24] Li X, Miao H, Henn A, Topham DJ, Wu H, Zand MS, et al Ki-67 expression reveals strong, transient influenza specific CD4 T cell responses after adult vaccination. Vaccine. 2012;30(31):4581–4. Epub 2012/05/05. 10.1016/j.vaccine.2012.04.059 22554464 PMC3858959

[25] Santos P, Lorena VM, Fernandes Ede S, Sales IR, Nascimento WR, Gomes Yde M, et al Gestation and breastfeeding in schistosomotic mothers differently modulate the immune response of adult offspring to postnatal Schistosoma mansoni infection. Memorias do Instituto Oswaldo Cruz. 2016;111(2):83–92. 10.1590/0074-02760150293 26872339 PMC4750447

[26] MATTHEW G, DARBY AC, DUNJA MRJDEN, MARION ROLOT, KATHERINE SMITH, CLAIRE MACKOWIAK, et al CUNNINGHAM, BENJAMIN G. DEWALS, FRANK BROMBACHER, WILLIAM G. C. HORSNELL. Pre-conception maternal helminth infection transfers via nursing long-lasting cellular immunity against helminths to offspring. Sci Adv. 2019;5 (5). 10.1126/sciadv.aav3058 31236458 PMC6587632

[27] Lostal Gracia MI, Larrad Mur L, Perez Gonzalez JM. [IgG subclasses: placental transfer in the full-term neonate and their evolution during the first 3 months of life]. An Esp Pediatr 1993;38(6):503–8. Epub 1993/06/01. . 8368678

[28] Okoko BJ, Wesumperuma LH, Ota MO, Pinder M, Banya W, Gomez SF, et al The influence of placental malaria infection and maternal hypergammaglobulinemia on transplacental transfer of antibodies and IgG subclasses in a rural West African population. J Infect Dis. 2001;184(5):627–32. Epub 2001/08/09. 10.1086/322808 . 11494168

[29] Pitcher-Wilmott RW, Hindocha P, Wood CB. The placental transfer of IgG subclasses in human pregnancy. Clin Exp Immunol 1980;41(2):303–8. Epub 1980/08/01. 7438556 PMC1537014

[30] Niewiesk S. Maternal antibodies: clinical significance, mechanism of interference with immune responses, and possible vaccination strategies. Front Immunol 2014;5:446 Epub 2014/10/04. 10.3389/fimmu.2014.00446 25278941 PMC4165321

[31] Tsutsui T, Yamamoto T, Hayama Y, Akiba Y, Nishiguchi A, Kobayashi S, et al Duration of maternally derived antibodies against Akabane virus in calves: survival analysis. J Vet Med Sci. 2009;71(7):913–8. Epub 2009/08/05. 10.1292/jvms.71.913 . 19652478

[32] McDonald EA, Cheng L, Jarilla B, Sagliba MJ, Gonzal A, Amoylen AJ, et al Maternal infection with Schistosoma japonicum induces a profibrotic response in neonates. Infect Immun. 2014;82(1):350–5. Epub 2013/10/30. 10.1128/IAI.01060-13 24166958 PMC3911825

[33] Al-Alwan M, Du Q, Hou S, Nashed B, Fan Y, Yang X, et al Follicular dendritic cell secreted protein (FDC-SP) regulates germinal center and antibody responses. J Immunol. 2007;178(12):7859–67. Epub 2007/06/06. 10.4049/jimmunol.178.12.7859 . 17548624

[34] Lacorcia M, Prazeres da Costa CU. Maternal Schistosomiasis: Immunomodulatory Effects With Lasting Impact on Allergy and Vaccine Responses Front Immunol 2018;9:2960 Epub 2019/01/09. 10.3389/fimmu.2018.02960 30619318 PMC6305477

[35] Labeaud AD, Malhotra I, King MJ, King CL, King CH. Do antenatal parasite infections devalue childhood vaccination? PLoS Negl Trop Dis 2009;3(5):e442 10.1371/journal.pntd.0000442 19478847 PMC2682196

[36] Seddon B, Tomlinson P, Zamoyska R. Interleukin 7 and T cell receptor signals regulate homeostasis of CD4 memory cells. Nat Immunol 2003;4(7):680–6. Epub 2003/06/17. 10.1038/ni946 . 12808452

[37] Anchi T, Tamura K, Furihata M, Satake H, Sakoda H, Kawada C, et al SNRPE is involved in cell proliferation and progression of high-grade prostate cancer through the regulation of androgen receptor expression. Oncol Lett. 2012;3(2):264–8. Epub 2012/06/29. 10.3892/ol.2011.505 22740892 PMC3362443

[38] Lan Y, Lou J, Hu J, Yu Z, Lyu W, Zhang B. Downregulation of SNRPG induces cell cycle arrest and sensitizes human glioblastoma cells to temozolomide by targeting Myc through a p53-dependent signaling pathway. Cancer Biol Med. 2020;17(1):112–31. Epub 2020/04/17. 10.20892/j.issn.2095-3941.2019.0164 32296580 PMC7142844

[39] Jia L, Bickel JS, Wu J, Morgan MA, Li H, Yang J, et al RBX1 (RING box protein 1) E3 ubiquitin ligase is required for genomic integrity by modulating DNA replication licensing proteins. The Journal of biological chemistry. 2011;286(5):3379–86. Epub 2010/12/01. 10.1074/jbc.M110.188425 21115485 PMC3030344

[40] Tolar P. Cytoskeletal control of B cell responses to antigens. Nat Rev Immunol 2017;17(10):621–34. Epub 2017/07/12. 10.1038/nri.2017.67 . 28690317

[41] Zhang X, Ren D, Guo L, Wang L, Wu S, Lin C, et al Thymosin beta 10 is a key regulator of tumorigenesis and metastasis and a novel serum marker in breast cancer. Breast Cancer Res. 2017;19(1):15 Epub 2017/02/10. 10.1186/s13058-016-0785-2 28179017 PMC5299657

[42] Ohkubo Y, Arima M, Arguni E, Okada S, Yamashita K, Asari S, et al A role for c-fos/activator protein 1 in B lymphocyte terminal differentiation. J Immunol. 2005;174(12):7703–10. Epub 2005/06/10. 10.4049/jimmunol.174.12.7703 . 15944271

[43] Yin Q, Wang X, McBride J, Fewell C, Flemington E. B-cell receptor activation induces BIC/miR-155 expression through a conserved AP-1 element. J Biol Chem 2008;283(5):2654–62. Epub 2007/12/01. 10.1074/jbc.M708218200 18048365 PMC2810639

[44] Claycombe KJ, Brissette CA, Ghribi O. Epigenetics of inflammation, maternal infection, and nutrition. J Nutr 2015;145(5):1109S–15S. Epub 2015/04/03. 10.3945/jn.114.194639 25833887 PMC4410493

[45] Bevan MJ. Understand memory, design better vaccines. Nat Immunol 2011;12(6):463–5. Epub 2011/05/19. 10.1038/ni.2041 21587308 PMC3303227

[46] Sallusto F, Lanzavecchia A, Araki K, Ahmed R. From vaccines to memory and back. Immunity 2010;33(4):451–63. Epub 2010/10/30. 10.1016/j.immuni.2010.10.008 21029957 PMC3760154

[47] Castellino F, Galli G, Del Giudice G, Rappuoli R. Generating memory with vaccination. Eur J Immunol 2009;39(8):2100–5. Epub 2009/07/29. 10.1002/eji.200939550 . 19637203

[48] Santos P, Lorena VM, Fernandes E, Sales IR, Albuquerque MC, Gomes Y, et al Maternal schistosomiasis alters costimulatory molecules expression in antigen-presenting cells from adult offspring mice. Experimental parasitology. 2014;141:62–7. 10.1016/j.exppara.2014.03.017 . 24657585

[49] Hara T, Shitara S, Imai K, Miyachi H, Kitano S, Yao H, et al Identification of IL-7-producing cells in primary and secondary lymphoid organs using IL-7-GFP knock-in mice. J Immunol. 2012;189(4):1577–84. Epub 2012/07/13. 10.4049/jimmunol.1200586 . 22786774

[50] Glatman Zaretsky A, Taylor JJ, King IL, Marshall FA, Mohrs M, Pearce EJ. T follicular helper cells differentiate from Th2 cells in response to helminth antigens. J Exp Med 2009;206(5):991–9. Epub 2009/04/22. 10.1084/jem.20090303 19380637 PMC2715032

[51] Korotkevich G, Sukhov V, Sergushichev A. Fast gene set enrichment analysis. bioRxiv. 2019:060012 10.1101/060012

[52] Subramanian A, Tamayo P, Mootha VK, Mukherjee S, Ebert BL, Gillette MA, et al Gene set enrichment analysis: a knowledge-based approach for interpreting genome-wide expression profiles. Proceedings of the National Academy of Sciences of the United States of America. 2005;102(43):15545–50. Epub 2005/10/04. 10.1073/pnas.0506580102 16199517 PMC1239896

