# Supplementary figures and images for: Maternal schistosomiasis impairs offspring Interleukin-4 production and B cell expansion
Source: PLoS Pathog. 2021 Feb 1;17(2):e1009260. doi: 10.1371/journal.ppat.1009260 (PMC7877777; doi:10.1371/journal.ppat.1009260)

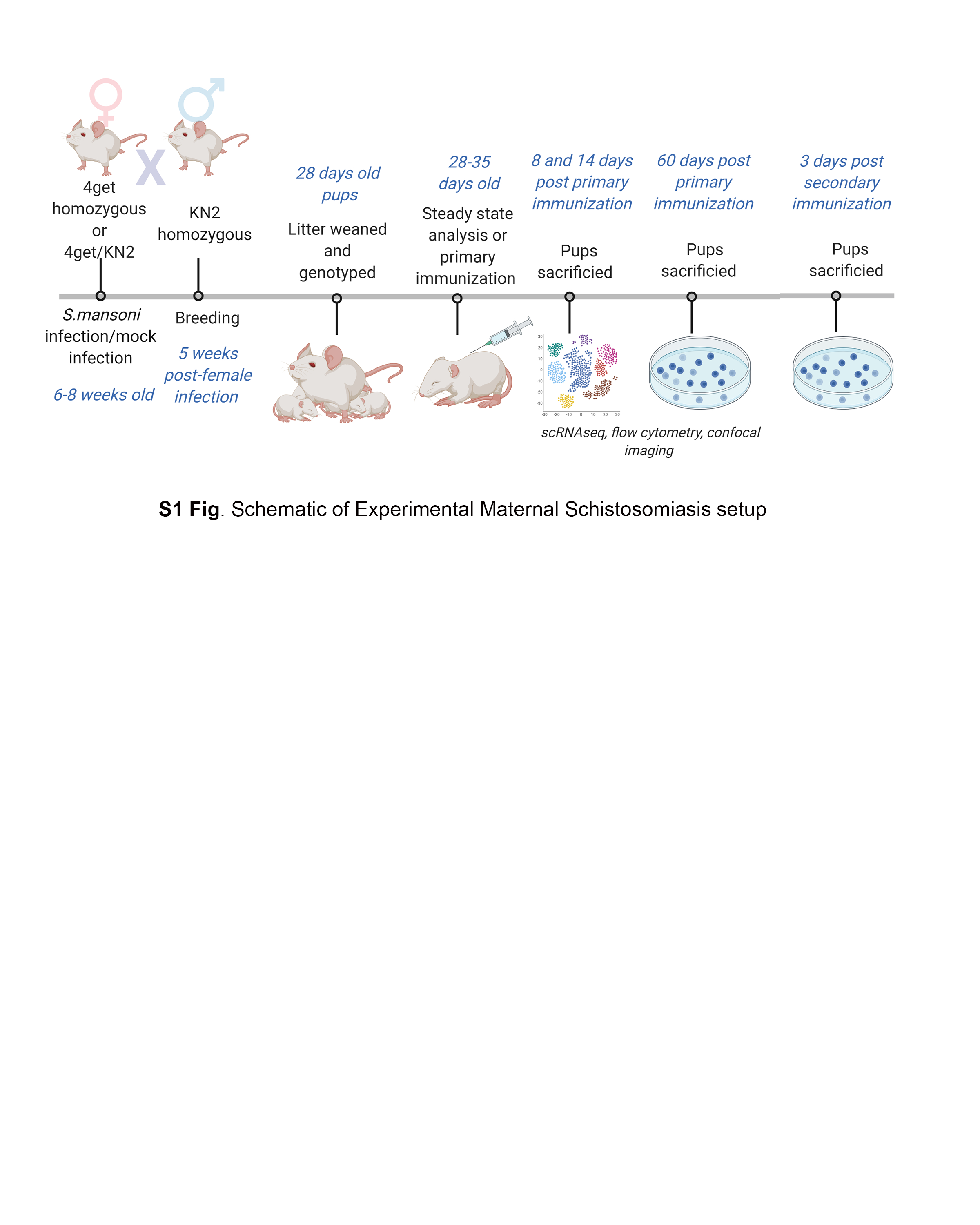

Supplement: S1 Fig — (TIF) [file ppat.1009260.s001.tif]

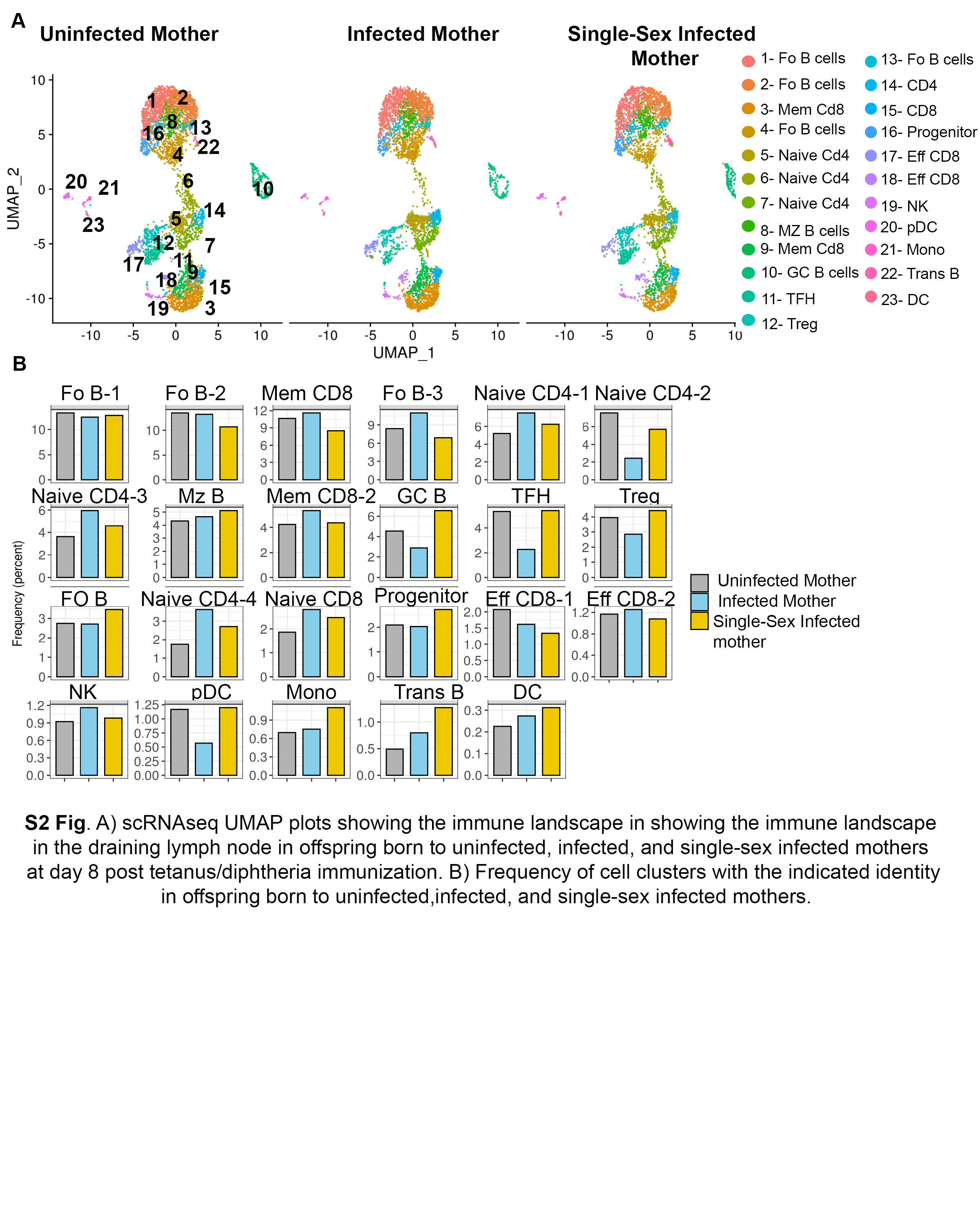

Supplement: S2 Fig — A) scRNAseq UMAP plots showing the immune landscape in showing the immune landscape in the draining lymph node in offspring born to uninfected, infected, and single-sex infected mothers at day 8 post tetanus/diphtheria immunization. B) Frequency of cell clusters with the indicated identity in offspring born to uninfected, infected, and single-sex infected mothers. (TIF) [file ppat.1009260.s002.tif]

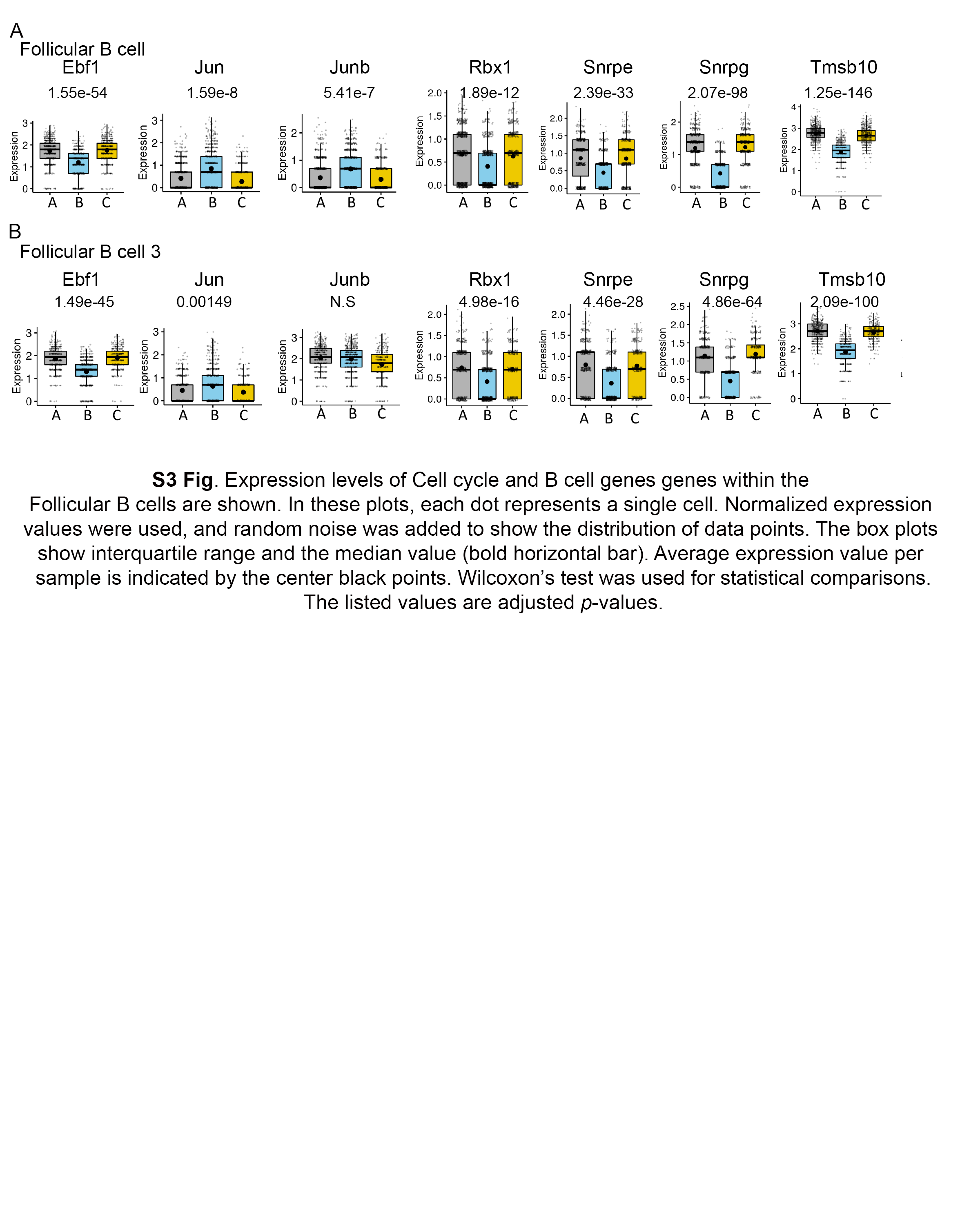

Supplement: S3 Fig — In these plots, each dot represents a single cell. Normalized expression values were used, and random noise was added to show the distribution of data points. The box plots show interquartile range and the median value (bold horizontal bar). Average expression value per sample is indicated by the center black points. Wilcoxon’s test was used for statistical comparisons. The listed values are adjusted p-values. (TIF) [file ppat.1009260.s003.tif]
